# Development and Validation of an Electronic Health Record–Based Algorithm for Identifying Patients With Long-Term Opioid Therapy: Cross-Sectional Study

**DOI:** 10.2196/76999

**Published:** 2025-12-10

**Authors:** Shu Huang, Tianze Jiao, Serena Jingchuan Guo, Jill A Star, Jie Xu, Jiang Bian, Debbie L Wilson, Amie J Goodin

**Affiliations:** 1 Department of Pharmaceutical Outcomes and Policy College of Pharmacy University of Florida Gainesville, FL United States; 2 Center for Drug Evaluation and Safety (CoDES) University of Florida Gainesville, FL United States; 3 Department of Psychiatry College of Medicine University of Florida Gainesville, FL United States; 4 Department of Health Outcomes and Biomedical Informatics College of Medicine University of Florida Gainesville, FL United States; 5 Department of Biostatistics and Health Data Science School of Medicine Indiana University Indianapolis, IN United States; 6 Regenstrief Institute Indianapolis, IN United States; 7 Shands Hospital University of Florida Gainesville, FL United States

**Keywords:** chronic pain, classification algorithm, electronic health records, opioid-related disorders, opioids, validation study

## Abstract

**Background:**

Health care providers must carefully monitor patients receiving long-term opioid therapy (LTOT) to minimize risks and maximize benefits. Yet, algorithms to support intervention during patient encounters are lacking, with accurate LTOT identification in routine care being the essential first step.

**Objective:**

This study aims to develop and validate an LTOT identification algorithm using electronic health record (EHR) data.

**Methods:**

In this cross-sectional study, we used 2016-2021 OneFlorida+ EHR data linked with Florida Medicaid claims to identify patients aged ≥18 years who received opioid prescriptions. The main outcome was the first LTOT episode in the algorithm development (2016-2018) and validation (2019-2021) periods. A Medicaid claims-based LTOT algorithm served as the reference standard, defined as ≥90 days of continuous opioid use with ≤15-day gaps. Given strong correlations among covariates, an elastic net regression model was applied to identify LTOT episodes in EHR data using patient characteristics, clinically relevant features, and medication use, and to evaluate the model’s classification performance. We randomly split the 2016-2018 cohort into development and internal validation datasets (2:1 ratio), stratified by LTOT incidence. External validation was performed using 2019-2021 data.

**Results:**

Among 64,206 eligible patients identified in 2016-2018 (mean age 35.7, SD 12.3 years; 51,421/64,206, 80.1% female), a total of 8899 (13.9%) had LTOT. Among 50,009 eligible patients identified in 2019-2021 (mean age 37.3, SD 12.5 years; 39,866/50,009, 79.7% female), a total of 6000 (12%) had LTOT. The model selected 29 out of 131 candidate features. Among 2967 individuals with LTOT in the 2016-2018 OneFlorida+ internal validation dataset, a total of 2176 (73.3%) individuals were identified in the top 3 deciles of risk scores. The model achieved a C-statistic of 0.83 (95% CI 0.82-0.84), with 73.4% (95% CI 71.8%-75%) sensitivity, 76.8% (95% CI 76.2%-77.4%) specificity, 33.8% (95% CI 33.1%-34.6%) precision, 76.3% (95% CI 75.8%-76.9%) accuracy, and an *F*_1_-score of 0.46. In the 2019-2021 OneFlorida+ external validation dataset, a total of 75.5% (4527/6000) individuals were correctly captured in the top 3 risk subgroups. The model achieved a C-statistic of 0.83 (95% CI 0.83-0.84), with 78.8% (95% CI 77.8%-79.9%) sensitivity, 73.3% (95% CI 72.9%-73.7%) specificity, 28.7% (95% CI 28.3%-29.1%) precision, 73.9% (73.6%-74.3%) accuracy, and an *F*_1_-score of 0.42.

**Conclusions:**

The EHR-based LTOT algorithm showed comparable accuracy to the claims-based reference and may support risk stratification and inform decision-making during clinical encounters.

## Introduction

Chronic noncancer pain is associated with various conditions that could significantly reduce patients’ quality of life and contribute to disability [1-3]. The reported prevalence of chronic pain in the general adult population ranges from 11% to 40% [4,5]. Approximately 10 million adults with chronic pain are prescribed long-term opioid therapy (LTOT) in the United States [6]. While LTOT effectively manages chronic pain, it carries risks of dependence, addiction, overdose, and even death [7]. Patients on LTOT for extended periods may develop tolerance and dependence, making it challenging to discontinue opioids [8]. Patients on LTOT with abrupt discontinuation of prescription opioids are vulnerable to withdrawal symptoms, such as psychological distress and worsening pain, which may lead to illicit drug use (eg, heroin) [9], and an increased risk of suicide [10-12]. Therefore, health care providers must carefully monitor patients on LTOT, regularly evaluate their pain levels and functional status, and adjust treatment plans as needed.

Identifying patients on LTOT within large-scale health care data can facilitate research on the effectiveness and safety of interventions aimed at opioid reduction, enabling the identification of candidates for alternative therapies to optimize pain management while mitigating opioid-related risks [13]. However, this can be challenging due to inconsistent LTOT definitions in the literature and variability in health care data structures, particularly within electronic health record (EHR) data, which often have limited longitudinal and comprehensive coverage across different health care systems. Recent literature more commonly adopts 90 days of supply as the cutoff in defining LTOT [14,15]. A recent systematic review found that 36% of LTOT definitions required a cumulative 3 months of opioid use [14]. Another scoping review identified 227 studies with different LTOT definitions, and half (49.8%) of them used a continuous 90-day opioid supply as a cutoff point to define LTOT [15]. This definition is also endorsed by the 2022 Centers for Disease Control and Prevention (CDC) clinical practice guideline [11]. In addition to inconsistent LTOT definitions, challenges exist when applying claims-based LTOT algorithms to EHR data. Only a few studies (20%) used EHR data for LTOT identification [15]. Carrell et al [16] developed an LTOT algorithm using structured EHR data, achieving a sensitivity of 58.2% at a precision of 57.2%. Unlike claims data that capture all prescriptions dispensed and reimbursed by the health plan, EHR data document patients’ clinical care records, including prescriptions ordered at specific health care systems where the patients visited [17]. Health care systems may use different EHR vendors (eg, EPIC and Cerner), and the completeness of EHR structured data fields varies due to differing data entry practices. Prescribing information (eg, quantity and days of supply) is often documented in unstructured clinical notes rather than structured data fields. This can lead to substantial missingness, making it difficult to directly apply claims-based LTOT algorithms to EHR data. These issues could hinder researchers’ ability to accurately calculate opioid doses or capture patients on LTOT when required information is missing from EHR structured data. Despite these limitations, EHR data are immediately available, enabling risk stratification during patient encounters, whereas claims often experience delays of months before being processed and adjudicated.

We aimed to develop and validate an EHR-based LTOT algorithm, with performance comparable to claims-based algorithms, using OneFlorida+ (OneFL) EHR linked to Florida Medicaid claims data.

## Methods

### Ethical Considerations

This study was exempted from review by the University of Florida Institutional Review Board (IRB202101897).

### Data Sources

This study used 2016-2021 OneFL EHR and Florida Medicaid linked data to identify adult patients who received opioids. The OneFL Data Trust is a centralized clinical data research network, containing approximately 75% of Floridians from 67 Florida counties [18,19]. OneFlorida was upgraded to OneFlorida+ in 2020, expanding the network from Florida to the southeastern United States [19]. Florida Medicaid is a part of OneFL Data Trust. As of November 2024, about 3.6 million Floridians were enrolled in Medicaid, representing approximately 15% of the state’s population [20].

The integrated data links Florida Medicaid claims and OneFL EHR data, with the potential to give a complete overview of patients’ medical and prescription history within the network.

### Study Design

**Figure 1:**
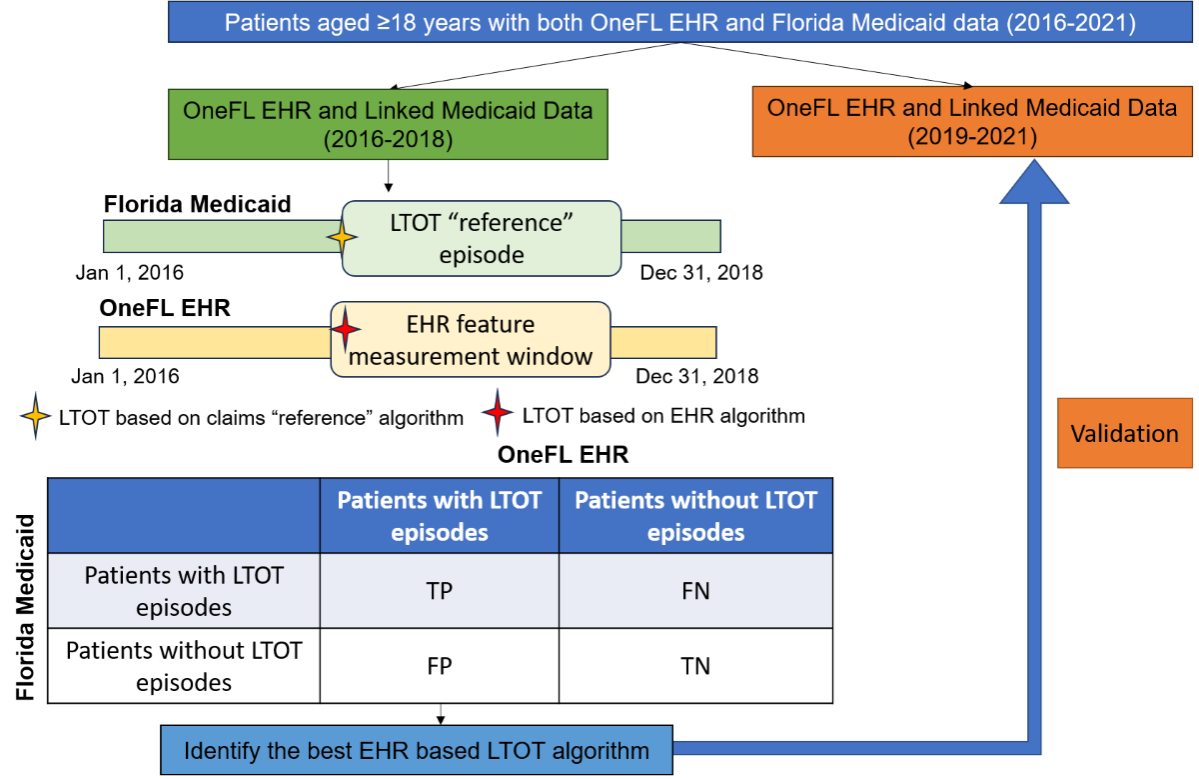
Validation study design schematic diagram. For patients with LTOT episodes, the index date was the initial date of the first LTOT episode; for patients with no LTOT episodes, the index date was the first date of the opioid prescription. EHR: electronic health record; LTOT: long-term opioid therapy; OneFL: OneFlorida+.

This study was a cross-sectional validation study (Figure 1). The patient population was people without cancer aged ≥18 years who received opioids between 2016 and 2021. We used RxNorm Concept Unique Identifier (RxCUI) codes to identify opioid medications in EHR prescribing data and National Drug Code (NDC) codes to identify opioid prescriptions in linked Florida Medicaid drug dispensing data. We excluded intravenous and injectable opioids typically administered in inpatient settings [21], as well as opioid medications indicated to treat cough or cold. Additionally, we excluded buprenorphine approved for opioid use disorders [22].

We used 2016-2018 data to develop and internally validate the EHR-based LTOT algorithm and then used data from more recent years (2019-2021) to externally validate its classification performance. The measured outcome was the first LTOT episode in the 2016-2018 and 2019-2021 periods. We applied a “reference standard” LTOT definition to Florida Medicaid claims prescription data to identify patients having a continuous opioid supply of ≥90 days within 180 days. To accommodate temporary lapses in prescription refills and gaps that may occur at the end of the study, we permitted a 15-day gap between the end date of the previous opioid prescription and the start date of the subsequent one [23].

We defined the index date as the initial date of an LTOT episode based on the “reference standard” (ie, the first date of the ≥90 days of continuous opioid supply within 180 days) in the primary analysis. For patients with multiple LTOT episodes, we used their first episode. For patients with no LTOT episodes, the index date was the first date of the opioid prescription in Florida Medicaid claims data. Beneficiaries were eligible for inclusion if they (1) had Florida Medicaid claims and OneFL EHR linked data, (2) were aged ≥18 years at the index date, (3) had no dual eligibility for Medicare and Medicaid (unable to capture prescriptions reimbursed by Medicare), (4) had ≥1 opioid prescription in Medicaid claims data, (5) had no malignant cancer diagnoses [24], (6) had no hospice service, (7) had continuous Medicaid enrollment for ≥3 months after the index date, and (8) had an index date on or before August 1, 2018, and August 1, 2021, separately (permitting enough follow-up time in EHR). We excluded patients with no visits recorded in their EHR 30 days before and 150 days after the index date (a total of 180 days to align with the “reference standard”).

We used a comprehensive list of factor candidates to develop the algorithm (Table 1), including patient demographics (eg, age, gender, race), any opioid and relevant substance use (eg, any prescription of muscle relaxants, benzodiazepines, gabapentinoids, and antidepressants), health services use (any inpatient, emergency department [ED], and outpatient visits), and comorbidities (eg, Elixhauser Comorbidity Index, depression, back pain, and chest pain) [16,25,26]. Patients on LTOT might have increased engagement with health care services for monitoring their opioid therapy, which may cause high medical costs [27-29]. The risk of complications (eg, overdose) can also lead to frequent ED visits [27]. Additionally, LTOT can interact with comorbidities, potentially affecting the management of these conditions and the overall health of the patient [30]. To account for the time lag in the claims data, the factors in EHR were measured 30 days before and 150 days after the index date.

**Table 1:** Potential factor candidates to develop electronic health record (EHR)–based long-term opioid therapy algorithms.

Category	Factor names
Demographics	age at index date, gender, race (ie, White, Black, others), ethnicity (ie, Hispanic, non-Hispanic, others), and metropolitan residence
Health services use	any hospitalization, ED^a^, and outpatient visits
Comorbidities	abdominal pain/hernia, adjustment disorder, aggression/impulsivity, AUD^b^, alcohol-induced mental disorders, anger issue, anxiety disorder, back pain, benzodiazepines overdose, bipolar disorder, cannabis use problems, caregiver issues, chest pain, cognitive confusion, delusional disorders, musculoskeletal disorders, dizziness, doctor/medical dissatisfaction, domestic violence experience or witness, emotional detachment, epilepsy, family history of mental and behavioral disorders, family related issues, fatigue, fibromyalgia, fractures/contusions/injuries, headache/migraine, heroin overdose, history of suicide-related events, HIV/AIDS, internal orthopedic device implant and graft, ischemic heart disease, kidney/gall stones, lack of appetite/appetite issue, lack of exercise, liver diseases, lost-time injuries or major surgeries, male genital disorders, maltreatment syndromes, menstrual/genital reproductive pain, mental and behavioral disorders due to psychoactive substance use, methadone treatment, miscellaneous mental health disorders, mood disorders, neck pain, neuropathies, non-opioid SUD^c^, osteoporosis pain, OUD^d^, opioid-related adverse events, opioid overdose, other nonorganic psychoses, other pains, other specified drug-induced mental disorders, pain catastrophizing, personality disorders, poisoning by other opiates and related narcotics, postoperative complications, PTSD^e^, problems related to lifestyle, problems related to social environment, psychiatric disorders, rheumatoid arthritis, respiratory diseases, schizophrenic disorders, status migraineurs, stroke, symptoms and signs involving emotional state, temporomandibular disorder pain, tinnitus, traumatic brain injury, urine drug test, and the Elixhauser Comorbidity Index and individual categories
Medication use	any use of opioids, antidepressants, antitussives, benzodiazepines, buprenorphine, gabapentinoids, muscle relaxants, naloxone, naltrexone, and multiple medication use (≥3 different medications other than listed above)
Opioid prescriber^f^	gender, and prescriber specialty (ie, primary care physicians, pain specialists, ED providers, surgeons, and others)

^a^ED: emergency department.

^b^AUD: alcohol use disorders.

^c^SUD: substance use disorders.

^d^OUD: opioid use disorders.

^e^PTSD: posttraumatic stress disorder.

^f^Opioid prescriber-level factors were included in the sensitivity analysis.

### Statistical Analysis

We described and compared patient demographic and clinical characteristics between the development and validation study cohorts. We excluded factors with high missingness (≥70%) from the analysis because they could give misleading associations, which may introduce bias and reduce model reliability. We then generated dummy variables for categorical factors (eg, race, ethnicity, and prescriber specialty) and imputed missing values to 0. For continuous factors (eg, age), we imputed missing values to the mean value. We randomly split the 2016-2018 cohort into two datasets stratified by the LTOT incident rate with a 2:1 ratio, using two-thirds as the training dataset for algorithm development and one-third for internal validation. We used Python (version 3.8; Python Software Foundation; eg, *sklearn* package) and applied the elastic net regression, a penalized regression, to the training dataset to identify LTOT. This model selects variables by shrinking the regression coefficients toward zero, which helps identify the most relevant factors and reduces the risk of overfitting [31]. It has the advantage of avoiding strong correlations between covariates by applying both L1 and L2 penalties, and it can handle high-dimensional data like EHR effectively. In addition, we compared the elastic net model performance with alternative models, including LR-L1, LR-L2, and a gradient-boosting machine. While C-statistics were similar across models, the elastic net model substantially outperformed the alternatives in sensitivity, which is critical for low-prevalence outcomes like LTOT (Table S1 in Multimedia Appendix 1). We performed hyperparameter tuning for the elastic net regression by varying parameters such as the L1-to-L2 penalty ratio and initial learning rate. Each parameter combination was used to train a model, and the best-performing model was selected based on C-statistics. We reported the selected factors, used the penalized coefficients directly, and applied the model to internal (2016-2018) and external (2019-2021) validation cohorts, where values of accuracy, sensitivity, specificity, precision, C-statistics, and *F*_1_-score were calculated (Table 2 and Table S2 in Multimedia Appendix 1). We divided the predicted LTOT risk scores generated by the training algorithm into 10 deciles. Each decile represents a range of risk scores, with the lowest decile indicating the lowest risk and the highest decile indicating the highest risk of LTOT. By applying these decile thresholds to internal and external validation cohorts, we classified and compared the risk profiles of patients in each dataset.

**Table 2:** Model performance.

Performance	OneFL^a^ EHR^b^ 2016-2018 internal validation (n=21,403)	OneFL EHR 2019-2021 external validation 1 (n=50,009)	OneFL EHR 2020-2021 external validation 2 (n=36,761)
Confusion Matrix (TP^c^-FP^d^-FN^e^-TN^f^), n	2179-4276-788-14,160	4730-11,760-1270-32,249	3846-8568-1049-23,298
LTOT^g^ rate, %	13.9	12	13.3
LTOT rate delta^h^, %	0	–1.9	–0.6
Sensitivity, % (95% CI)	73.4 (71.8-75)	78.8 (77.8-79.9)	78.6 (77.4-79.7)
Specificity, % (95% CI)	76.8 (76.2-77.4)	73.3 (72.9-73.7)	73.1 (72.6-73.6)
Precision, % (95% CI)	33.8 (33.1-34.6)	28.7 (28.3-29.1)	31 (30.5-31.5)
Accuracy, % (95% CI)	76.3 (75.8-76.9)	73.9 (73.6-74.3)	73.8 (73.4-74.3)
AUROC^i^, (95% CI)	0.83 (0.82-0.84)	0.83 (0.83-0.84)	0.83 (0.83-0.84)
AUPRC^j^, (95% CI)	0.24 (0.23-0.24)	0.21 (0.20-0.21)	0.22 (0.22-0.23)
*F*_1_-score	0.46	0.42	0.44

^a^OneFL: OneFlorida+.

^b^EHR: electronic health record.

^c^TP: true positive (the number of cases correctly identified as LTOT).

^d^FP: false positive (the number of cases incorrectly identified as LTOT).

^e^FN: false negative (the number of cases incorrectly identified as non-LTOT).

^f^TN: true negative (the number of cases correctly identified as non-LTOT).

^g^LTOT: long-term opioid therapy.

^h^LTOT rate deltas represent the changes in LTOT rates relative to the OneFL EHR 2016-2018 training data (n=42,803; LTOT rate=13.9%)

^i^AUROC: area under the receiver operating characteristic curve.

^j^AUPRC: area under the precision-recall curve.

We conducted 4 sensitivity analyses. First, we applied other commonly used claims-based LTOT algorithms (ie, ≥120 continuous days of supply in 180 days) as the “reference standard” [15,32]. Second, we added provider-level factors to the primary model. Primary opioid prescriber characteristics included gender and prescriber specialty (ie, primary care physicians, pain specialists, ED providers, surgeons, and others). Third, to offset the potential policy impacts of Florida House Bill 21 [33], we applied the primary model to more recent years (2020-2021) to test robustness. Fourth, studies have found racial treatment completion disparities [34]; therefore, we checked for any race discrimination (White vs Black patients) by examining the false negative (FN) and false positive (FP) rates by race.

## Results

**Figure 2:**
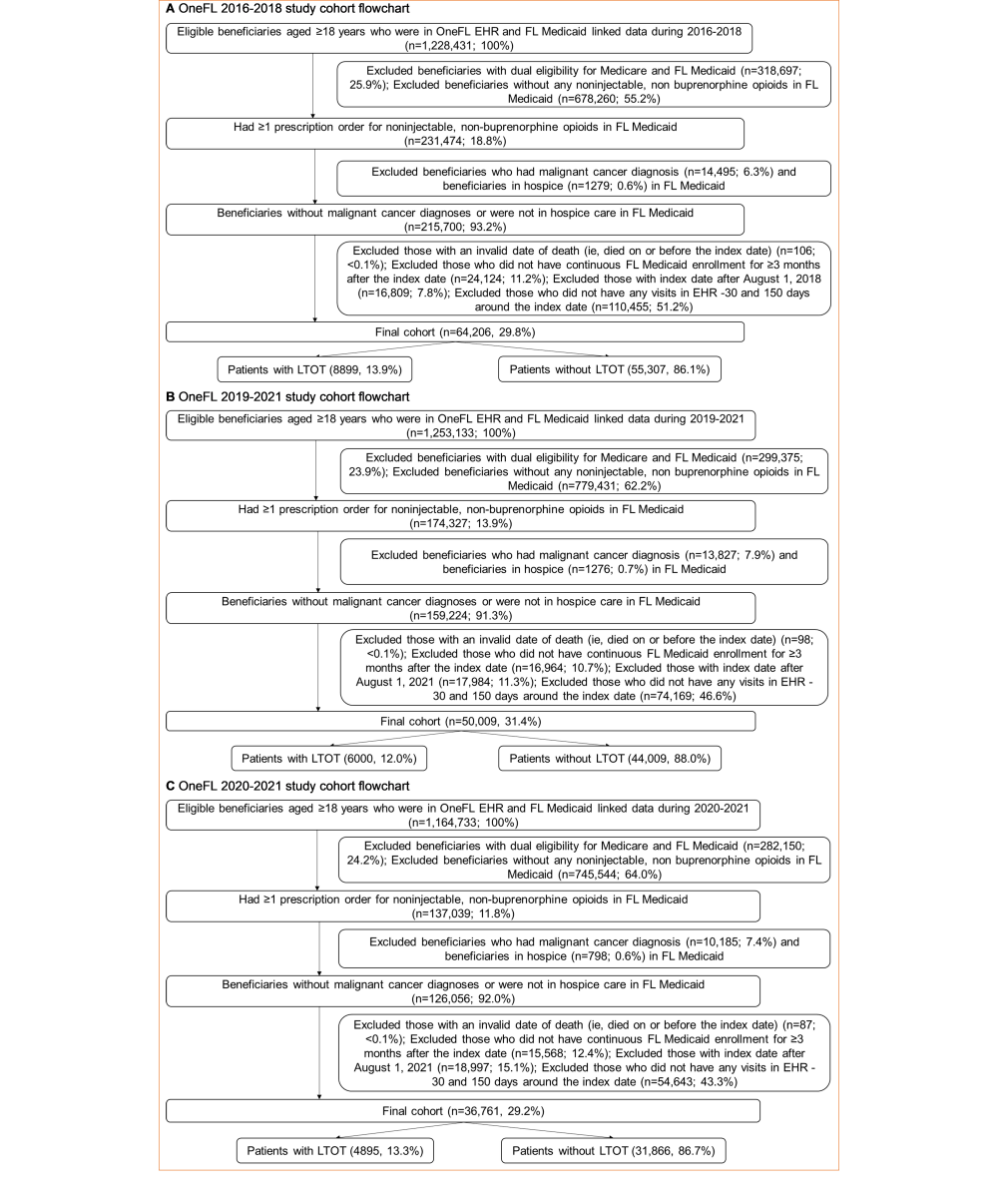
Flowcharts of OneFL study cohorts. EHR: electronic health record; FL: Florida; LTOT: long-term opioid therapy; OneFL: OneFlorida+.

Of 1,228,431 beneficiaries in the 2016-2018 dataset, a total of 64,206 adult patients without cancer were included, with 42,803 (66.7%) for algorithm development and 21,403 (33.3%) for internal validation (Figure 2). Most patients were female (51,421/64,206, 80.1%), White (33,635/64,206, 52.4%), and non-Hispanic (48,211/64,206, 75.1%; Table 3). Overall, a total of 8899 (13.9%) patients had ≥1 LTOT episode in the 2016-2018 Medicaid claims data. Of 1,253,133 beneficiaries in the 2019-2021 dataset, we included 50,009 patients for external validation. Most patients were female (39,866/50,009, 79.7%), White (25,838/50,009, 51.7%), and non-Hispanic (37,398/50,009, 74.8%). Compared to the overall 2016-2018 cohort, patients in the 2019-2021 cohort had a lower incidence of LTOT (12% vs 13.9%), were older (mean age: 37.3, SD 12.5 years vs 35.7, SD 12.3 years) and sicker (Elixhauser Comorbidity Index: 1.4, SD 1.9 vs 1.2, SD 1.8); were more likely to have outpatient visits (35,280/50,009, 70.5% vs 42,585/64,206, 66.3%), inpatient visits (14,746/50,009, 29.5% vs 15,883/64,206, 24.7%), anxiety (6610/50,009, 13.2% vs 6825/64,206, 10.6%), depression (4372/50,009, 8.7% vs 4850/64,206, 7.6%), mood disorders (5840/50,009, 11.7% vs 6422/64,206, 10%), musculoskeletal disorders (15,313/50,009, 30.6% vs 18,738/64,206, 29.2%), obesity (9422/50,009, 18.8% vs 8827/64,206, 13.7%), and opioid-related adverse events (2671/50,009, 5.3% vs 3006/64,206, 4.7%); were more likely to be prescribed gabapentinoids (4938/50,009, 9.9% vs 4086/64,206, 6.4%), muscle relaxants (5238/50,009, 10.5% vs 5717/64,206, 8.9%), and naloxone (897/50,009, 1.8% vs 155/64,206, 0.2%); and were less likely to use nonintravenous opioids (15,555/50,009, 31.1% vs 30,522/64,206, 47.5%). We observed similar findings in the more recent 2020-2021 cohort.

**Table 3:** Selected patient characteristics.

Patient characteristics				OneFL^a^ EHR^b^ 2016-2018						OneFL EHR 2019-2021		OneFL EHR 2020-2021
				Overall (n=64,206)		Training (n=42,803)		Internal validation (n=21,403)		External validation 1 (n=50,009)		External validation 2 (n=36,761)
LTOT^c^ incidence rate (90-day) in Florida Medicaid claims, n (%)				8899 (13.9)		5932 (13.9)		2967 (13.9)		6000 (12)		4895 (13.3)
LTOT incidence rate (120-day) in Florida Medicaid claims, n (%)				7250 (11.3)		4827 (11.3)		2423 (11.3)		5121 (10.2)		4343 (11.8)
**Sociodemographics**												
	Age at index date (years), mean (SD)		35.7 (12.3)		35.7 (12.3)		35.7 (12.3)		37.3 (12.5)		37.5 (12.8)	
	**Gender, n (%)**											
		Female	51,421 (80.1)		34,227 (80.0)		17,194 (80.3)		39,866 (79.7)		29,068 (79.1)	
		Male	12,785 (19.9)		8576 (20.0)		4209 (19.7)		10,143 (20.3)		7693 (20.9)	
	**Race, n (%)**											
		White	33,635 (52.4)		22,414 (52.4)		11,221 (52.4)		25,838 (51.7)		19,028 (51.8)	
		Black	24,945 (38.9)		16,611 (38.8)		8334 (38.9)		19,254 (38.5)		14,033 (38.2)	
		Others or unknown	5626 (8.8)		3778 (8.8)		1848 (8.6)		4917 (9.8)		3700 (10.1)	
	**Ethnicity, n (%)**											
		Non-Hispanic	48,211 (75.1)		32,165 (75.1)		16,046 (75.0)		37,398 (74.8)		27,508 (74.8)	
		Hispanic	15,598 (24.3)		10,374 (24.2)		5224 (24.4)		12,113 (24.2)		8,807 (24.0)	
		Others or unknown	397 (0.6)		264 (0.6)		133 (0.6)		498 (1.0)		446 (1.2)	
	Metropolitan area		60,518 (94.3)		40,372 (94.3)		20,146 (94.1)		46,935 (93.9)		34,445 (93.7)	
**Health services use, n (%)**												
	≥1 inpatient visit		15,883 (24.7)		10,539 (24.6)		5344 (25)		14,746 (29.5)		10,693 (29.1)	
	≥1 emergency department visit		25,102 (39.1)		16,727 (39.1)		8375 (39.1)		16,157 (32.3)		11,373 (30.9)	
	≥1 outpatient visit		42,585 (66.3)		28,406 (66.4)		14,179 (66.2)		35,280 (70.5)		26,294 (71.5)	
**Medication use, n (%)**												
	Any antidepressant use		5846 (9.1)		3912 (9.1)		1934 (9.0)		4833 (9.7)		3552 (9.7)	
	Any antitussive use		244 (0.4)		163 (0.4)		81 (0.4)		66 (0.1)		35 (0.1)	
	Any benzodiazepine use		4620 (7.2)		3042 (7.1)		1578 (7.4)		2207 (4.4)		1416 (3.9)	
	Any buprenorphine use		76 (0.1)		53 (0.1)		23 (0.1)		84 (0.2)		54 (0.1)	
	Any gabapentinoid use		4086 (6.4)		2733 (6.4)		1353 (6.3)		4938 (9.9)		3724 (10.1)	
	Any muscle relaxant use		5717 (8.9)		3868 (9.0)		1849 (8.6)		5238 (10.5)		3847 (10.5)	
	Any naloxone use		155 (0.2)		109 (0.3)		46 (0.2)		897(1.8)		533 (1.4)	
	Any naltrexone use		36 (0.1)		23 (0.1)		13 (0.1)		22 (<0.1)		19 (0.1)	
	Any nonintravenous opioid use		30,522 (47.5)		20,306 (47.4)		10,216 (47.7)		15,555 (31.1)		10,386 (28.3)	
	Multiple medication use (≥3)		28,998 (45.2)		19,333 (45.2)		9665 (45.2)		20,866 (41.7)		14,792 (40.2)	
**Comorbidities**												
	Elixhauser Index, mean (SD)		1.2 (1.8)		1.2 (1.8)		1.2 (1.8)		1.4 (1.9)		1.4 (2.0)	
	Alcohol use disorder, n (%)		1,152 (1.8)		725 (1.7)		427 (2.0)		936 (1.9)		672 (1.8)	
	Anxiety, n (%)		6825 (10.6)		4575 (10.7)		2250 (10.5)		6610 (13.2)		5055 (13.8)	
	Depression, n (%)		4850 (7.6)		3285 (7.7)		1565 (7.3)		4372 (8.7)		3296 (9.0)	
	Hypertension, n (%)		12,664 (19.7)		8460 (19.8)		4204 (19.6)		11,382 (22.8)		8500 (23.1)	
	Lost-time injuries or major surgeries, n (%)		12,370 (19.3)		8290 (19.4)		4080 (19.1)		10,144 (20.3)		7,494 (20.4)	
	Mood disorder, n (%)		6422 (10.0)		4352 (10.2)		2070 (9.7)		5840 (11.7)		4393 (12.0)	
	Musculoskeletal disorders, n (%)		18,738 (29.2)		12,583 (29.4)		6155 (28.8)		15,313 (30.6)		11,436 (31.1)	
	Obesity, n (%)		8827 (13.7)		5903 (13.8)		2924 (13.7)		9422 (18.8)		7201 (19.6)	
	Opioid-related adverse events (selected)^d^, n (%)		3006 (4.7)		1992 (4.7)		1014 (4.7)		2671 (5.3)		2053 (5.6)	
	Opioid overdose^e^, n (%)		127 (0.2)		81 (0.2)		46 (0.2)		77 (0.2)		56 (0.2)	
	Respiratory diseases, n (%)		12,594 (19.6)		8401 (19.6)		4193 (19.6)		9994 (20.0)		7364 (20.0)	
	SUDs^f^ other than opioid overdose, n (%)		10,986 (17.1)		7275 (17.0)		3711 (17.3)		8686 (17.4)		6229 (16.9)	
	Abdominal pain, n (%)		11,807 (18.4)		7811 (18.2)		3996 (18.7)		9128 (18.3)		6756 (18.4)	
	Back pain, n (%)		8779 (13.7)		5951 (13.9)		2828 (13.2)		6686 (13.4)		4969 (13.5)	
	Chest pain, n (%)		4906 (7.6)		3285 (7.7)		1621 (7.6)		3750 (7.5)		2743 (7.5)	
	Headache or migraine, n (%)		5647 (8.8)		3761 (8.8)		1886 (8.8)		4369 (8.7)		3253 (8.8)	

^a^OneFL: OneFlorida+.

^b^EHR: electronic health record.

^c^LTOT: long-term opioid therapy.

^d^Opioid-related adverse events (selected) includes opioid-induced sleep disorders, mental disorders, and respiratory issues.

^e^Opioid overdose includes nonfatal poisoning by heroin, methadone, opiates, and related narcotics.

^f^SUD: substance use disorders (SUDs other than opioid overdose include nonfatal poisoning and disorders caused by substances such as alcohol, cannabis, etc).

**Figure 3:**
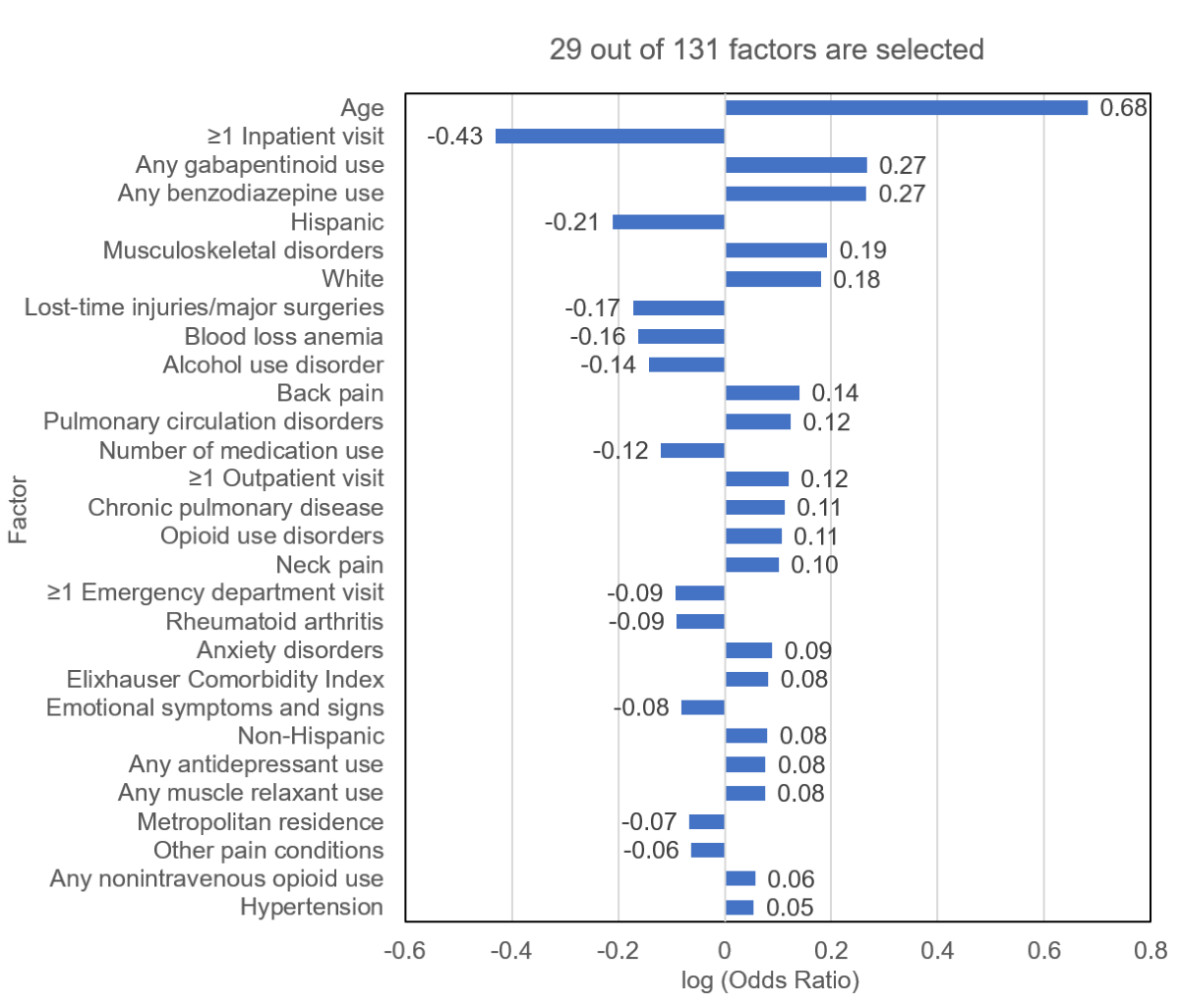
Selected factors using elastic net regression in OneFlorida+.

**Figure 4:**
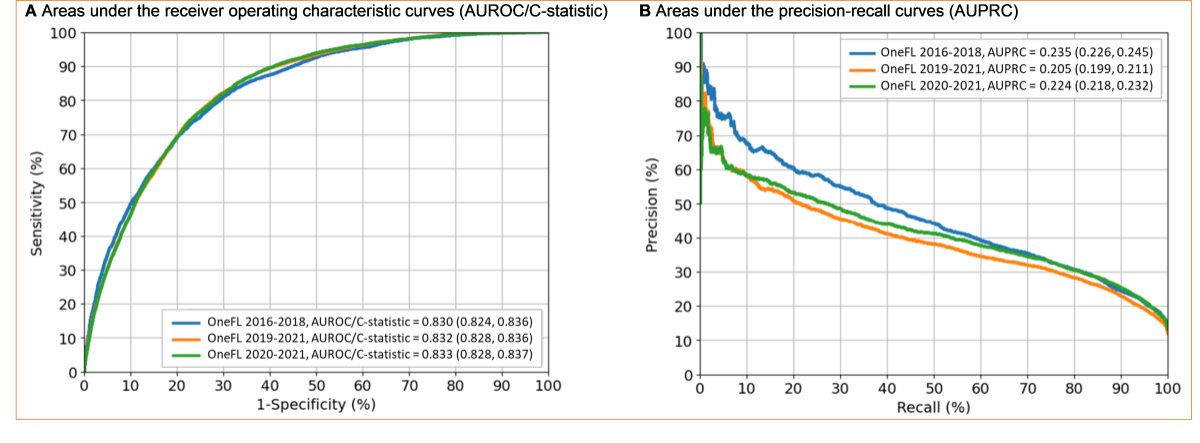
Performance matrix for identifying patients with long-term opioid therapy using elastic net regression in OneFlorida+.

Our elastic net model selected 29 out of 131 factors (Figure 3), including age, ≥1 inpatient visits, gabapentinoid use, benzodiazepine use, Hispanic ethnicity, musculoskeletal disorders, and White race. The algorithm performed well in validation datasets. For the 2016-2018 internal validation dataset, the C-statistic was 0.83 (95% CI 0.82-0.84); for the 2019-2021 external validation dataset, it was 0.83 (95% CI 0.83-0.84); and for the 2020-2021 external validation dataset, it was 0.83 (95% CI 0.83-0.84) (Figure 4). For the 2016-2018 internal validation dataset, the sensitivity, specificity, precision, accuracy, and *F*_1_-score were 73.4% (95% CI 71.8%-75%), 76.8% (95% CI 76.2%-77.4%), 33.8% (95% CI 33.1%-34.6%), 76.3% (95% CI 75.8%-76.9%), and 0.46, respectively. For the 2019-2021 external validation dataset, the corresponding metrics were 78.8% (95% CI 77.8%-79.9%), 73.3% (95% CI 72.9%-73.7%), 28.7% (95% CI 28.3%-29.1%), 73.9% (95% CI 73.6%-74.3%), and 0.42, respectively. For the 2020-2021 external validation dataset, the corresponding metrics were 78.6% (95% CI 77.4%-79.7%), 73.1% (95% CI 72.6%-73.6%), 31.0% (95% CI 30.5%-31.5%), 73.8% (95% CI 73.4%-74.3%), and 0.44, respectively.

**Figure 5:**
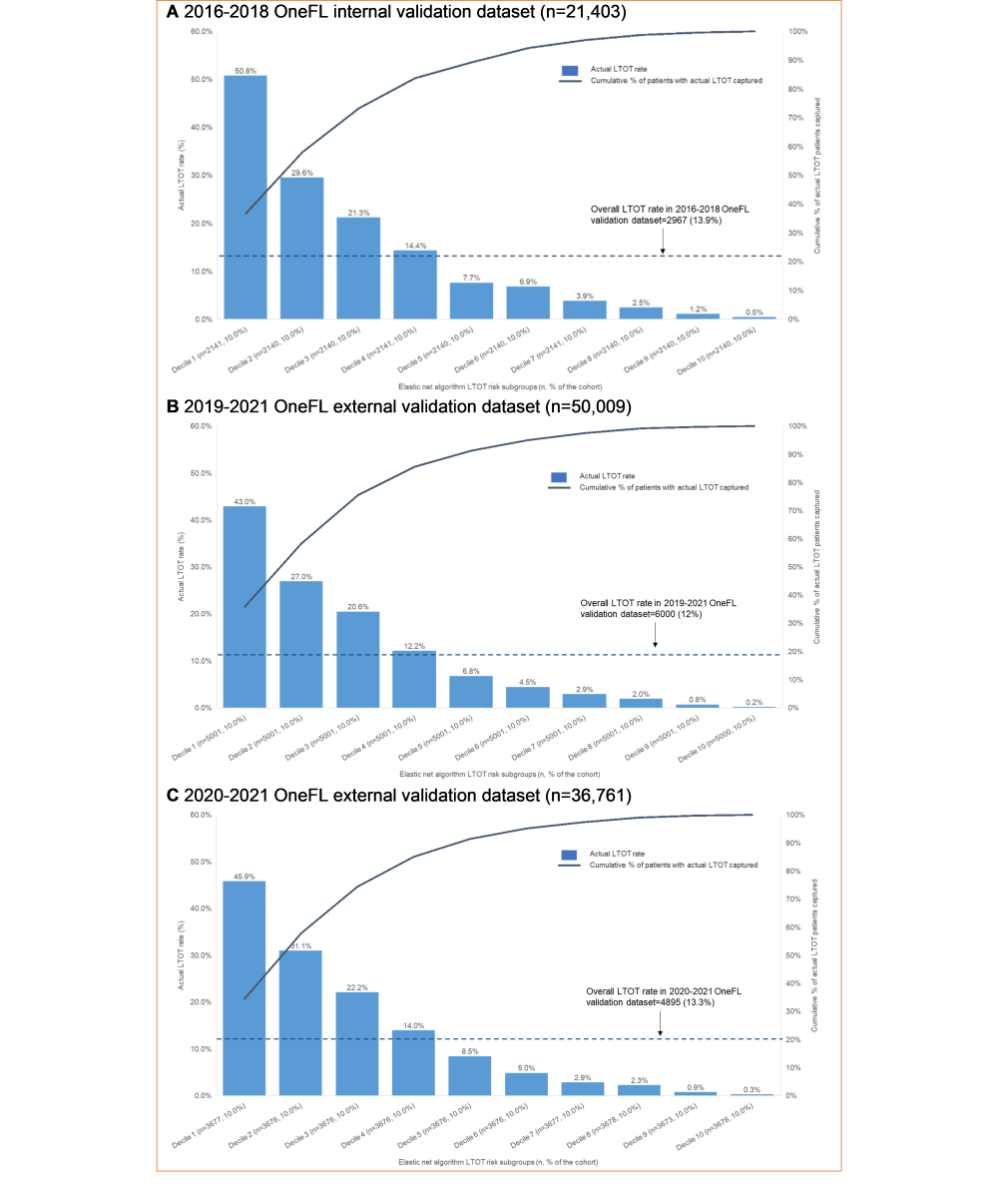
Patients with long-term opioid therapy identified by risk subgroups in the OneFlorida+ internal and external validation datasets using the elastic net regression model. LTOT: long-term opioid therapy.

**Figure 6:**
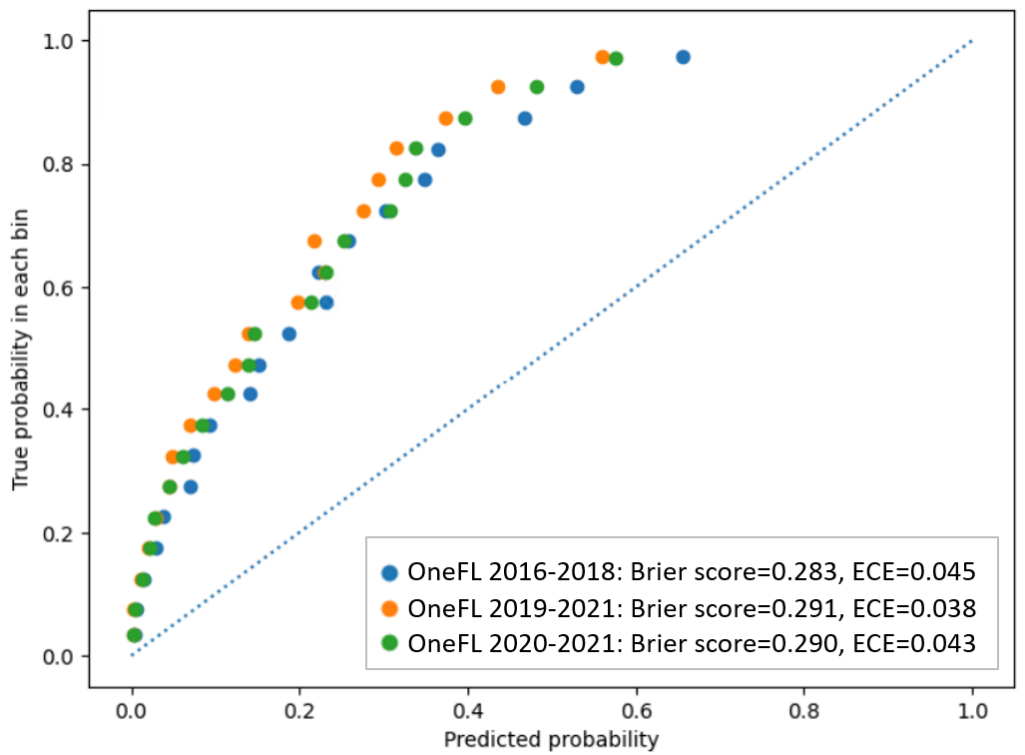
Calibration plot for the OneFlorida+ internal and external validation datasets using the elastic net regression model (for 20 population bins of equal size). ECE: expected calibration error.

Figure 5 depicts the actual LTOT rate for individuals in the validation datasets using risk score thresholds derived from the 2016-2018 OneFL training dataset. The thresholds of the risk scores to identify a patient’s LTOT risk subgroup are: Decile 1 (highest risk subgroup; risk score≥80.6), Decile 2 (64.1≤risk score<80.6), Decile 3 (50.3≤risk score<64.1), Decile 4 (39.6≤risk score<50.3), Decile 5 (31.4≤risk score<39.6), Decile 6 (24.9≤risk score<31.4), Decile 7 (19.5≤risk score<24.9), Decile 8 (14.3≤risk score<19.5), Decile 9 (9.0≤risk score<14.3), and Decile 10 (risk score<9.0). The highest-risk subgroup (Decile 1: n=2141) had a precision of 50.8%. Among 2967 individuals with LTOT in the 2016-2018 internal validation dataset, a total of 2176 (73.3%) were captured in the top 3 deciles of risk scores. The 4th-10th decile subgroups had lower LTOT rates (0.5%-14.4%). The LTOT rate in the 2019-2021 external validation dataset was lower than in the internal validation dataset (12% vs 13.9%). The highest-risk subgroup (Decile 1: n=5001) had a precision of 43%. Of 6000 individuals with LTOT in the 2019-2021 OneFL external validation dataset, a total of 4527 (75.5%) were captured in the top 3 risk subgroups. The 4th-10th risk subgroups had lower LTOT rates (0.2%-12.2%) than the top 3 decile subgroups. Similarly, in 2020-2021 OneFL external validation analyses, the LTOT rate was slightly lower than in the internal validation dataset (13.3% vs 13.9%). The highest-risk subgroup (Decile 1: n=3677) had a precision of 45.9%. Of 4895 individuals with LTOT in the 2020-2021 external validation dataset, a total of 3647 (74.5%) were identified in the top 3 risk subgroups. The 4th-10th risk subgroups had lower LTOT rates (0.3%-14%). The calibration plot (Figure 6) shows that our model tends to overestimate the LTOT risk across subgroups when comparing predicted and observed risks, as it predicts a probability of LTOT higher than the actual event probability. If the model is perfectly calibrated, the points would align along the diagonal line. For instance, if the predicted probability of LTOT is 0.8, the actual observed frequency of the event should occur approximately 80% of the time in the bin.

**Figure 7:**
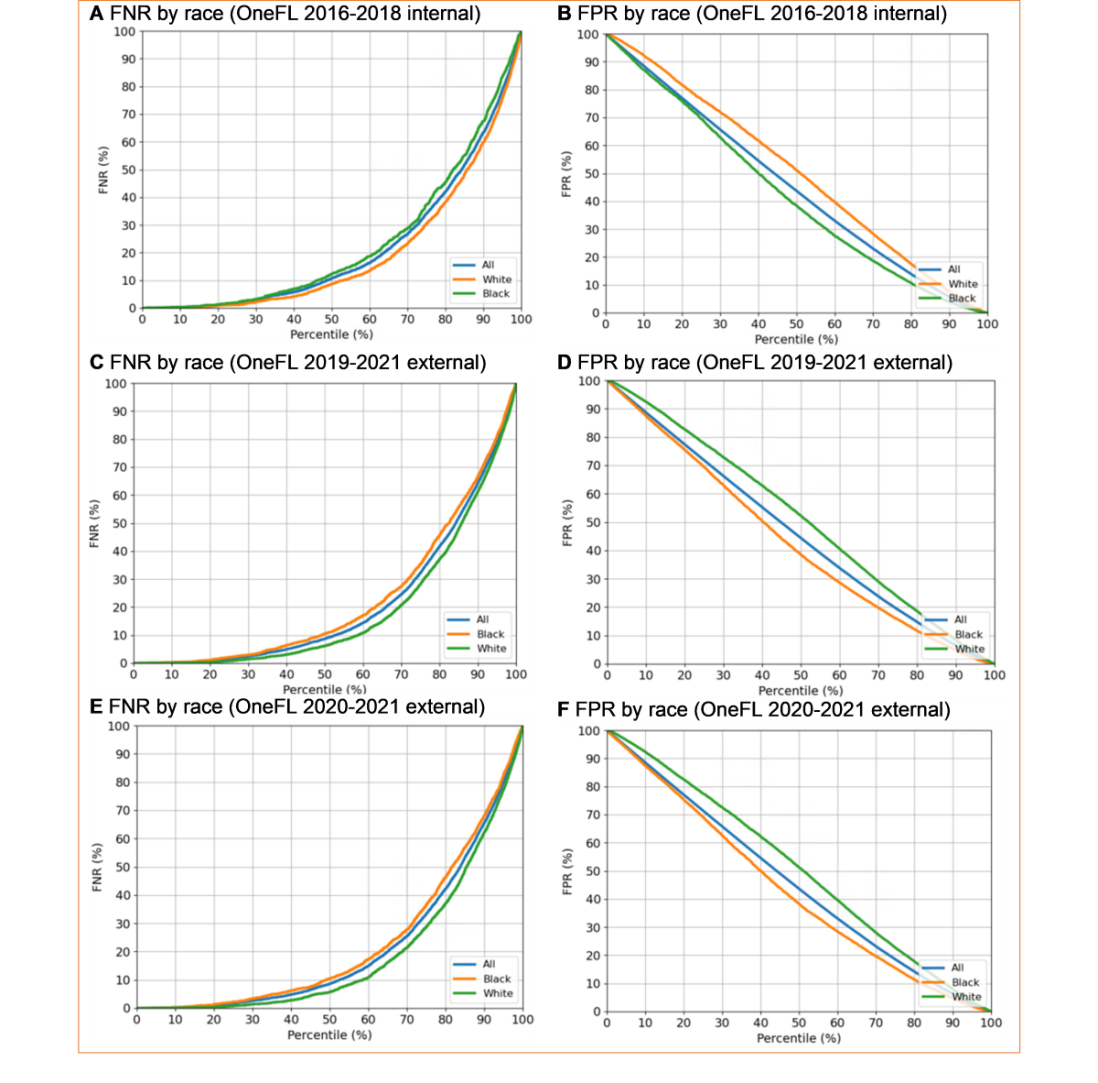
Race discrimination check for the OneFlorida+ internal and external validation datasets using the elastic net regression model. FNR: false negative rate, FPR: false positive rate.

The sensitivity analyses yielded results consistent with the main findings. When using 120 days of supply as the LTOT cutoff, we reached similar C-statistics as the primary analyses. For the 2016-2018 dataset, the C-statistic was 0.83 (95% CI 0.82-0.84); for the 2019-2021 dataset, it was 0.84 (95% CI 0.84-0.85). Moreover, after adding prescriber-level factors to the model, the C-statistics remained high: 0.83 (95% CI 0.83-0.84) for the 2016-2018 dataset and 0.85 (95% CI 0.84-0.85) for the 2019-2021 dataset. This new algorithm identified 27 out of 138 factors. Primary care physicians, pain specialists, ED providers, and obesity were included in this new model, replacing Elixhauser Comorbidity Index, hypertension, nonintravenous opioid use, antidepressant use, muscle relaxant use, and other pain conditions. Third, we observed no race discrimination in LTOT (Figure 7). The FN and FP rates between Black and White patients were similar in internal and external validation datasets.

## Discussion

### Principal Results

We developed and validated an EHR-based algorithm with high C-statistics, sensitivity, specificity, and accuracy for identifying LTOT in adult patients without cancer with opioid prescriptions. The algorithm highlighted key risk factors for LTOT, which can be applied in related research to identify patients on LTOT.

The patient populations in the 2016-2018 development and the 2019-2021 validation datasets differed significantly. Compared with the 2016-2018 cohort, we observed a lower LTOT incidence rate and a generally sicker patient population in the 2019-2021 OneFL EHR dataset, possibly due to the Florida policy changes and the COVID-19 pandemic. Opioid supply restriction policies were implemented in Florida (eg, the supply limit and the Prescription Drug Monitoring Program mandate), as well as insurer and health-system initiatives that occurred simultaneously, leading to fewer patients on LTOT in the EHR system. For example, local primary care providers who had previously managed patients on LTOT were encouraged to refer them to pain management specialists. The COVID-19 pandemic and quarantine policies also limited patient visits to clinics. Due to these factors, fewer patients were included in the 2019-2021 cohort and were more likely to have visits for more severe conditions such as hypertension and musculoskeletal disorders. These patients were also more likely to suffer from psychological pain, including anxiety, depression, and mood disorders. Patients in the newer dataset represented a higher-risk population than those in the older dataset. Despite that, the model still performed well.

Although our algorithm showed high discrimination (C-statistics >0.80), the relatively low LTOT rate resulted in low precision (<40%), which could increase FP and overestimate the benefits. However, the model’s sensitivity was fairly high (>70%), making it effective in detecting patients on LTOT. The low precision affects the *F*_1_-score, making the balance between precision and sensitivity relatively low. This means that while the model could be effective at identifying patients on LTOT (high sensitivity), it also generates many FP (low precision). However, in highly imbalanced clinical outcomes like LTOT, models with lower *F*_1_-scores can still provide meaningful risk stratification and clinical decision support, particularly given the serious risks associated with opioid misuse. Therefore, our model would perform better in a population with a higher LTOT incidence rate. Reporting additional measures, such as sensitivity and the *F*_1_-score, gave a more thorough evaluation and helped decision-makers better understand the benefits and limitations of the model when implementing it.

A recent systematic review identified age, race, arthritis, pain conditions, mental health disorders, and substance use disorders as the most common LTOT risk factors, all of which were selected in our EHR-based algorithm [14]. Furthermore, adding prescriber-level factors may improve the algorithm’s performance. However, since not all EHR systems include prescriber information, these factors were supplementary. We tested the robustness of the model by varying the LTOT “reference standard” definitions, using ≥90 and ≥120 days of consecutive opioid use. However, applying a longer cutoff for opioid use (eg, ≥180 days) requires extended follow-up to capture a sufficient number of LTOT episodes. Caution should be exercised when the algorithm is used in an environment with limited follow-up time or high rates of missing data.

### Strengths and Limitations

This study has several strengths. First, we leveraged a linked EHR-claims dataset, incorporated a comprehensive list of factors, and demonstrated robust validation across different time periods to identify LTOT. The approach was less costly and time-consuming than medical chart review, which has been the standard method for validating health outcomes of interest in EHR [35]. It addressed the challenge of identifying patients on LTOT in EHR structured data due to different data entry practices. Second, the EHR-based algorithm was evaluated against the “reference standard” using multiple performance metrics. Our model showed high C-statistics (>0.80), indicating it can correctly discriminate patients on LTOT from patients not on LTOT. The sensitivity and specificity were also good (>0.73), meaning that the model is effective at detecting the presence of LTOT. In research, correctly identifying patients on LTOT can help study trends, adverse events, and potential complications associated with LTOT, informing clinical guidelines and policies. In clinical practice, it would allow clinics to implement enhanced monitoring strategies, such as regular screenings for opioid misuse, checking for opioid-related adverse events, and tracking prescription patterns. It can also enhance clinical decision-making by applying tailored care plans for patients on LTOT. This study developed an EHR-based model for LTOT ascertainment, rather than a predictive model, using EHR features measured over a timeframe mostly aligned with the linked claims data. Although not all pre-index EHR features were used for classification, the model may provide valuable insights that can inform future prospective efforts to identify high-risk patients before LTOT initiation, supporting targeted interventions and prevention strategies. Third, we validated the EHR-based algorithm using internal data and external data from more recent years. This study showed that an LTOT algorithm developed in one state’s EHR could effectively translate to later time periods in the same US state with a population of different patient characteristics, addressing a significant concern regarding the generalizability of LTOT algorithms.

This study has several limitations. First, prescriptions paid out-of-pocket or through alternative payment methods (eg, cash) were not captured in the claims data, potentially resulting in an underestimated LTOT rate. Second, the significant missingness among variables in the EHR data (eg, days of supply, quantity) could introduce misclassification. The absence of key information may lead to inaccurate identification of patients on LTOT or incorrect risk assessments, affecting the overall reliability of the algorithm. Consequently, the model’s performance may be compromised, reducing its accuracy in real-world applications. Third, we could not capture factors from unstructured data (eg, clinical notes), which often hold valuable clinical insights. However, much of this information was already inferred from the structured EHR data, including diagnoses, procedures, and prescription records. Incorporating unstructured data in future studies could provide additional context and potentially enhance the model’s performance. Likewise, integrating regional-level factors, such as zip code–level opioid-related mortality rates, could further enhance predictive accuracy. Future research may also benefit from combining geospatial information with public health surveillance data to strengthen model validity and applicability. Fourth, this algorithm might not generalize to non-OneFL patient populations or states, as it is based on a linked database including mainly Floridians. However, the modeling framework could be replicated using datasets from other states or national databases to validate external applicability. Finally, the precision was low due to the relatively low rate of LTOT. However, our risk-stratified approach appeared effective in validation datasets. Over 73% of patients on LTOT were captured in the top 3 deciles of risk scores. Focusing on high-risk subgroups enables identification of patients most at risk for LTOT.

### Conclusions

The EHR-based LTOT algorithm derived from OneFL data performed well in internal validation with data from the same years and external validation with data from more recent years. The algorithm has the potential to be applied to related research to identify patients on LTOT and help clinics with opioid management and decision-making in time.

## Data Availability

Data used in this study are available to researchers under the terms of a data use agreement from the OneFlorida Clinical Research Network. The datasets generated or analyzed during this study are available from the corresponding author on reasonable request.

## Appendix

Multimedia Appendix 1 Supplementary tables (competitive models' performance, performance metrics, and ICD-10-CM and CPT codes).

## Abbreviations

CDC: Centers for Disease Control and Prevention
ED: emergency department
EHR: electronic health record
FN: false negative
FP: false positive
LTOT: long-term opioid therapy
NDC: National Drug Code
OneFL: OneFlorida+
RxCUI: RxNorm Concept Unique Identifier
