# Supplementary material for: Development and Validation of an Electronic Health Record–Based Algorithm for Identifying Patients With Long-Term Opioid Therapy: Cross-Sectional Study
Source: J Med Internet Res. 2025 Dec 10;27:e76999. doi: 10.2196/76999 (PMC12739455; doi:10.2196/76999)
Supplement: Multimedia Appendix 1 [file jmir_v27i1e76999_app1.docx]

**Multimedia Appendix 1**

**Table S1.** Competitive Models' performance, Using OneFL EHR 2016-2018 Internal Validation Dataset (N=21,403)

**Table S2.** Performance Metrics Used in the Study

**Table S3.** ICD-10-CM and CPT Codes Used in the Study

**Table S1. Competitive Models' performance, Using OneFL EHR 2016-2018 Internal Validation Dataset (N=21,403)**

| Performance | Logistic Regression (L1) | Logistic Regression (L2) | Gradient-boosting Machine |
| --- | --- | --- | --- |
| Confusion Matrix (TP-FP-FN-TN) | 707-466-2260-17,970 | 705-468-2262-17,968 | 781-363-2186-18,073 |
| LTOT rate | 13.9% | 13.9% | 13.9% |
| Sensitivity | 23.8% [22.3%, 25.4%] | 23.8% [22.2%, 25.3%] | 26.3% [24.8%, 28.0%] |
| Specificity | 97.5% [97.2%, 97.7%] | 97.5% [97.2%, 97.7%] | 98.0% [97.8%, 98.2%] |
| Precision | 60.3% [57.6%, 62.9%] | 60.1% [57.4%, 62.7%] | 68.3% [65.7%, 70.8%] |
| Accuracy | 87.3% [86.8%, 87.7%] | 87.2% [86.8%, 87.7%] | 88.1% [87.7%, 88.5%] |
| AUROC | 0.83 [0.82, 0.84] | 0.83 [0.83, 0.84] | 0.85 [0.85, 0.86] |
| AUPRC | 0.25 [0.24, 0.26] | 0.25 [0.24, 0.26] | 0.28 [0.27, 0.30] |
| F1 Score | 0.34 | 0.34 | 0.38 |

**Abbreviations:** AUROC: area under the receiver operating characteristic curve, AUPRC: area under the precision-recall curve, LTOT: long-term opioid therapy, FN: false negative (the number of cases incorrectly identified as non-LTOT), FP: false positive (the number of cases incorrectly identified as LTOT), TN: true negative (the number of cases correctly identified as non-LTOT), TP: true positive (the number of cases correctly identified as LTOT).

Compared with our elastic net model, the alternative models achieve comparable AUROC/C-statistic (>0.83); however, their F1 scores (<0.4) and sensitivities (<30%) are low, making them ineffective for detecting LTOT patients, which is a critical consideration given the low prevalence of LTOT.

**Table S2. Performance Metrics Used in the Study**

| Metrics | Equation | Description |
| --- | --- | --- |
| Accuracy | $Accuracy=\frac{TP+TN}{TP+TN+FP+FN}$ | Measures the proportion of correctly identifying LTOT cases and non-cases |
| AUROC | *Sensitivity vs. (1- Specificity)* | Quantifies the overall performance of a classification model based on its receiver operating characteristic curve |
| AUPRC | *Precision vs. Sensitivity* | Quantifies the overall performance of a classification model based on its precision-recall curve |
| Precision | $Precision=\frac{TP}{TP+FP}$ | Measures the proportion of predicted LTOT cases that truly have LTOT |
| Sensitivity (Recall) | $Sensitivity=\frac{TP}{TP+FN}$ | Measures the proportion of true LTOT cases that are correctly identified by the algorithm |
| Specificity | $Specificity=\frac{TN}{TN+FP}$ | Measures the proportion of non-LTOT cases that are correctly identified by the algorithm |
| F1 score | $F1=\frac{2TP}{2TP+FP+FN}$ | The harmonic mean of precision and sensitivity |

**Abbreviations:** AUROC: area under the receiver operating characteristic curve, AUPRC: area under the precision-recall curve, LTOT: long-term opioid therapy, FN: false negative (the number of cases incorrectly identified as non-LTOT), FP: false positive (the number of cases incorrectly identified as LTOT), TN: true negative (the number of cases correctly identified as non-LTOT), TP: true positive (the number of cases correctly identified as LTOT).

**Table S3. ICD-10-CM and CPT Codes Used in the Study**

| **Factor** | **ICD-10-CM and CPT Codes** |
| --- | --- |
| Alcohol use disorder | F10.1x, F10.2x |
| Anxiety disorders | F06.4, F40.x-F42.x, F43.0, F44.9, F45.8, F48.8, F48.9, F99, R45.7, F93.8 |
| Back pain | M40.00, M40.209, M40.299, M40.4, M40.5, M41.00, M41.20, M41.30, M41.80, M41.9, M43.00, M43.10, M43.27, M43.28, M43.8X9, M45.9, M46.00, M46.1, M46.40, M46.45, M46.47, M46.5, M46.8, M46.9, M47.14, M47.15, M47.16, M48.00, M48.04, M48.06, M48.08, M48.1, M48.2, M48.3, M48.9, M49.8, M51.04, M51.05, M51.06, M51.2, M51.3, M51.36, M51.37, M51.46, M51.47, M51.8, M51.9, M53.2X7, M53.2X8, M53.3, M53.9, M54.08, M54.14, M54.15, M54.16, M54.17, M54.30, M54.5, M54.6, M54.89, M54.9, M96.1, M96.2, M96.3, M96.4, M96.5, M99.02, M99.03, M99.04, M99.83, M99.84, Q76.0, Q76.1, Q76.419, Q76.49, S12.9XXA, S22.009A, S23.101A, S23.3XXA, S23.8XXA, S23.9XXA, S32.009A, S32.10XA, S32.2XXA, S33.101A, S33.2XXA, S33.5XXA, S33.6XXA, S33.8XXA, S33.9XXA |
| Blood loss anemia | D50.0 |
| Chronic pulmonary disease | I27.8, I27.9, J40.x-J47.x, J60.x-J67.x, J68.4, J70.1, J70.3 |
| Emotional symptoms and signs | R45.x |
| Hypertension | I10.x-I13.x, I15.x |
| Lost-time injuries/major surgeries | S00.x-S99.x, T07.x, T14.x-T28.x, T30.x-T34.x, T36.x-T88.x, Y83.x, Y84.x |
|  | **CPT codes:** 11008, 11970, 19101, 19105, 19110, 19112, 19120, 19125, 19126, 19300, 19301, 19302, 19303, 19305, 19306, 19307, 19316, 19318, 19324, 19325, 19328, 19330, 19340, 19342, 19350, 19355, 19357, 19361, 19364, 19366, 19367, 19368, 19369, 19370, 19371, 19380, 20101, 20102, 21603, 22220, 22222, 22224, 22532, 22533, 22548, 22551, 22554, 22556, 22558, 22586, 22590, 22595, 22600, 22610, 22612, 22630, 22633, 22800, 22802, 22804, 22808, 22810, 22812, 22856, 22857, 22861, 22862, 22867, 23615, 23616, 23630, 23670, 23680, 23900, 23920, 24515, 24516, 24545, 24546, 24575, 24579, 24586, 24587, 24635, 24665, 24666, 24685, 24900, 24920, 24930, 24931, 25337, 25515, 25525, 25526, 25545, 25574, 25575, 25607, 25608, 25609, 25652, 25900, 25905, 25909, 25920, 25922, 25924, 25927, 25929, 25931, 26235, 26236, 26551, 26910, 26951, 26952, 27125, 27130, 27132, 27134, 27137, 27138, 27177, 27178, 27179, 27181, 27236, 27244, 27245, 27248, 27254, 27269, 27280, 27290, 27295, 27438, 27440, 27441, 27442, 27443, 27445, 27446, 27447, 27486, 27487, 27506, 27507, 27511, 27513, 27514, 27519, 27535, 27536, 27540, 27590, 27591, 27592, 27598, 27758, 27759, 27766, 27769, 27784, 27792, 27814, 27822, 27823, 27826, 27827, 27828, 27829, 27880, 27881, 27882, 27884, 27886, 27888, 27889, 28124, 28126, 28160, 28800, 28805, 28810, 28820, 28825, 31300, 31360, 31365, 31367, 31368, 31370, 31375, 31380, 31382, 31390, 31395, 31400, 31420, 31551, 31552, 31553, 31554, 31560, 31561, 31580, 31584, 31587, 31590, 31591, 31592, 31599, 31770, 31775, 32096, 32097, 32100, 32110, 32120, 32124, 32140, 32141, 32151, 32200, 32215, 32220, 32225, 32310, 32320, 32440, 32442, 32445, 32480, 32482, 32484, 32486, 32488, 32491, 32501, 32503, 32504, 32505, 32506, 32507, 32540, 32553, 32607, 32608, 32609, 32651, 32652, 32655, 32658, 32659, 32661, 32662, 32663, 32666, 32667, 32668, 32669, 32670, 32671, 32672, 32800, 32815, 32905, 32906, 32940, 32960, 33020, 33025, 33030, 33031, 33050, 33120, 33130, 33202, 33203, 33206, 33207, 33208, 33212, 33213, 33214, 33215, 33216, 33217, 33218, 33220, 33221, 33222, 33223, 33224, 33225, 33226, 33227, 33228, 33229, 33230, 33231, 33233, 33234, 33235, 33236, 33237, 33238, 33240, 33241, 33243, 33244, 33249, 33250, 33251, 33254, 33255, 33256, 33257, 33258, 33259, 33261, 33262, 33263, 33264, 33265, 33266, 33267, 33268, 33269, 33270, 33271, 33272, 33273, 33300, 33305, 33310, 33315, 33365, 33366, 33390, 33391, 33404, 33405, 33406, 33410, 33411, 33412, 33413, 33414, 33415, 33416, 33417, 33420, 33422, 33425, 33426, 33427, 33430, 33440, 33460, 33463, 33464, 33465, 33468, 33470, 33471, 33474, 33475, 33476, 33478, 33496, 33509, 33510, 33511, 33512, 33513, 33514, 33516, 33533, 33534, 33535, 33536, 33542, 33545, 33548, 33600, 33602, 33608, 33610, 33611, 33612, 33615, 33617, 33619, 33641, 33645, 33647, 33660, 33665, 33670, 33675, 33676, 33677, 33681, 33684, 33688, 33692, 33694, 33697, 33702, 33710, 33720, 33722, 33732, 33735, 33736, 33737, 33770, 33774, 33776, 33780, 33782, 33783, 33786, 33813, 33814, 33889, 33920, 33927, 33928, 33929, 33935, 33945, 33975, 33976, 33977, 33978, 33979, 33980, 34830, 34831, 34832, 35081, 35082, 35091, 35092, 35102, 35103, 35301, 35390, 35501, 35506, 35508, 35509, 35510, 35511, 35512, 35515, 35516, 35518, 35521, 35522, 35523, 35525, 35533, 35556, 35558, 35566, 35570, 35571, 35583, 35585, 35587, 35600, 35616, 35621, 35623, 35650, 35654, 35656, 35661, 35666, 35671, 35686, 35840, 36800, 36810, 36815, 36818, 36819, 36820, 36821, 36825, 36830, 36832, 36833, 36838, 38100, 38101, 38102, 38115, 38120, 38308, 38542, 38720, 38724, 39000, 39010, 39200, 39220, 39501, 39503, 39540, 39541, 39545, 39560, 39561, 41135, 41140, 41145, 41155, 42420, 42425, 42426, 43117, 43118, 43121, 43122, 43286, 43287, 43288, 43320, 43332, 43333, 43360, 43496, 43500, 43501, 43502, 43520, 43605, 43610, 43611, 43620, 43621, 43622, 43631, 43632, 43633, 43634, 43640, 43641, 43644, 43645, 43770, 43771, 43772, 43773, 43774, 43775, 43800, 43810, 43820, 43825, 43840, 43842, 43843, 43845, 43846, 43847, 43848, 43850, 43855, 43860, 43865, 43870, 43880, 43886, 43887, 43888, 44005, 44010, 44020, 44021, 44025, 44110, 44111, 44120, 44125, 44126, 44127, 44130, 44137, 44140, 44141, 44143, 44144, 44145, 44146, 44147, 44150, 44151, 44155, 44156, 44157, 44158, 44160, 44180, 44186, 44187, 44188, 44202, 44204, 44205, 44206, 44207, 44208, 44210, 44211, 44212, 44213, 44227, 44300, 44310, 44312, 44314, 44316, 44320, 44322, 44340, 44345, 44346, 44602, 44603, 44604, 44605, 44615, 44620, 44625, 44626, 44640, 44650, 44700, 44800, 44820, 44850, 44900, 44950, 44955, 44960, 44970, 44979, 45110, 45111, 45112, 45113, 45114, 45116, 45119, 45120, 45121, 45126, 45136, 45395, 45397, 45562, 45563, 47010, 47015, 47100, 47120, 47122, 47125, 47130, 47135, 47140, 47141, 47142, 47300, 47350, 47360, 47361, 47362, 47370, 47371, 47379, 47380, 47381, 47400, 47420, 47425, 47460, 47480, 47562, 47563, 47564, 47570, 47600, 47605, 47610, 47612, 47620, 47700, 47701, 47711, 47712, 47715, 47720, 47721, 47740, 47741, 47760, 47765, 47780, 47785, 47800, 47802, 47900, 48000, 48001, 48020, 48100, 48105, 48120, 48140, 48145, 48146, 48148, 48150, 48152, 48153, 48154, 48155, 48160, 48500, 48510, 48520, 48540, 48545, 48548, 49000, 49002, 49010, 49013, 49014, 49020, 49040, 49060, 49203, 49204, 49205, 49215, 49220, 49250, 49255, 49320, 49321, 49324, 49325, 49326, 49402, 49412, 49419, 49421, 49425, 49426, 49491, 49492, 49495, 49496, 49500, 49501, 49505, 49507, 49520, 49521, 49525, 49550, 49553, 49555, 49557, 49560, 49561, 49565, 49566, 49570, 49572, 49580, 49582, 49585, 49587, 49590, 49600, 49605, 49606, 49610, 49611, 49650, 49651, 49652, 49653, 49654, 49655, 49656, 49657, 49659, 49900, 49905, 49906, 50010, 50020, 50040, 50045, 50060, 50065, 50070, 50075, 50120, 50125, 50130, 50135, 50205, 50220, 50225, 50230, 50234, 50236, 50240, 50250, 50280, 50290, 50320, 50340, 50360, 50365, 50380, 50400, 50405, 50541, 50542, 50543, 50545, 50546, 50547, 50548, 50549, 51925, 55705, 55810, 55812, 55815, 55821, 55831, 55840, 55842, 55845, 55866, 57307, 58150, 58152, 58180, 58200, 58210, 58240, 58260, 58262, 58263, 58267, 58270, 58275, 58280, 58285, 58290, 58291, 58292, 58293, 58294, 58541, 58542, 58543, 58544, 58548, 58550, 58552, 58553, 58554, 58570, 58571, 58572, 58573, 58575, 58660, 58661, 58662, 58679, 58720, 58740, 58800, 58805, 58820, 58822, 58825, 58900, 58920, 58925, 58940, 58943, 58950, 58951, 58952, 58953, 58954, 58956, 58960, 58970, 59100, 59510, 59514, 59515, 59525, 59618, 59620, 59622, 59857, 60000, 60200, 60210, 60212, 60220, 60225, 60240, 60252, 60254, 60260, 60270, 60271, 60280, 60281, 60500, 60502, 60505, 60512, 61105, 61107, 61108, 61120, 61140, 61150, 61151, 61154, 61156, 61210, 61250, 61253, 61304, 61305, 61312, 61313, 61314, 61315, 61320, 61321, 61322, 61323, 61330, 61333, 61340, 61343, 61345, 61458, 61460, 61510, 61512, 61514, 61516, 61518, 61519, 61520, 61521, 61522, 61524, 61526, 61530, 61531, 61533, 61534, 61535, 61536, 61537, 61538, 61539, 61540, 61541, 61543, 61544, 61545, 61546, 61548, 61566, 61567, 61570, 61571, 61575, 61576, 61580, 61581, 61582, 61583, 61584, 61585, 61586, 61590, 61591, 61592, 61595, 61598, 61600, 61601, 61605, 61606, 61607, 61608, 61615, 61616, 61618, 61619, 61680, 61682, 61684, 61686, 61690, 61692, 61697, 61698, 61700, 61702, 61703, 61705, 61708, 61710, 61711, 61720, 61735, 61736, 61737, 61750, 61751, 61760, 61770, 61850, 61860, 61863, 61867, 61870, 61880, 62000, 62005, 62010, 62100, 62120, 62121, 62160, 62161, 62163, 62164, 62165, 62180, 62190, 62192, 62194, 62200, 62201, 62220, 62223, 62225, 62230, 62256, 62258, 62287, 62351, 62380, 63001, 63003, 63005, 63011, 63012, 63015, 63016, 63017, 63020, 63030, 63035, 63040, 63042, 63045, 63046, 63047, 63048, 63050, 63051, 63055, 63056, 63064, 63075, 63077, 63081, 63082, 63085, 63086, 63087, 63088, 63090, 63091, 63101, 63102, 63103, 63170, 63172, 63173, 63180, 63182, 63185, 63190, 63191, 63194, 63195, 63196, 63197, 63198, 63199, 63200, 63250, 63251, 63252, 63265, 63266, 63267, 63268, 63270, 63271, 63272, 63273, 63275, 63276, 63277, 63278, 63280, 63281, 63282, 63283, 63285, 63286, 63287, 63290, 63709, 63740, 64746, 69150, 69155, 0051T, 0052T, 0053T, 0202T, 0219T, 0220T, 0221T, 0571T, 0572T, 0573T, 0574T, 0585T, 0586T |
| Malignancy | C00.x-C96.x |
| Musculoskeletal disorders | M67.47, M70.30, D48.1, M02.00, M02.20, M02.9, M05.00, M05.10, M05.30, M05.60, M06.1, M06.4, M06.9, M08.00, M08.3, M08.40, M11.20, M11.219, M11.229, M11.239, M11.249, M11.259, M11.269, M11.279, M11.28, M11.29, M11.80, M11.819, M11.829, M11.839, M11.849, M11.859, M11.869, M11.879, M11.88, M11.89, M11.9, M12.00, M12.10, M12.119, M12.129, M12.139, M12.149, M12.159, M12.169, M12.179, M12.18, M12.19, M12.50, M12.80, M12.80, M12.8x, M12.9, M13.0, M13.1x, M13.8x, M14.60, M14.80, M15.0, M15.1, M15.2, M15.3, M15.8, M15.9, M16.10, M16.7, M16.9, M17.10, M17.5, M17.9, M18.9, M19.019, M19.029, M19.039, M19.049, M19.079, M19.219, M19.229, M19.239, M19.249, M19.279, M19.90, M19.91, M19.91, M19.93, M19.93, M20.009, M20.019, M20.029, M20.039, M20.099, M20.10, M20.10, M20.20, M20.30, M20.40, M20.5X9, M20.60, M21.029, M21.059, M21.069, M21.129, M21.159, M21.169, M21.339, M21.40, M21.5x, M21.61, M21.62, M21.6X9, M21.759, M21.769, M21.80, M21.839, M21.859, M21.869, M21.90, M21.939, M21.959, M21.969, M22.40, M23.009, M23.202, M23.205, M23.2x, M23.305, M23.319, M23.329, M23.339, M23.359, M23.369, M23.40, M23.50, M23.8X9, M24.x, M24.20, M25.x, M25.70, M25.729, M32.10, M33.20, M33.90, M34.0, M34.1, M34.9, M35.00, M35.01, M35.3, M35.5, M35.7, M35.8, M35.9, M36.2, M36.3, M36.4, M40.00, M40.10, M40.209, M40.299, M40.40, M40.50, M41.00, M41.20, M41.30, M41.40, M41.50, M41.80, M41.9, M42.00, M42.10, M43.00, M43.10, M43.27, M43.28, M43.6, M43.8X9, M43.8X9, M43.8X9, M45.9, M46.00, M46.1, M46.20, M46.30, M46.40, M46.45, M46.47, M46.80, M46.90, M47.10, M47.12, M47.14, M47.16, M47.812, M47.814, M47.817, M47.819, M48.00, M48.02, M48.04, M48.06, M48.08, M48.10, M48.20, M48.30, M48.4, M48.50XA, M48.9, M49.80, M50.00, M50.20, M50.30, M50.80, M50.90, M51.0x, M51.2x, M51.3x, M51.4x, M51.8x, M51.9, M51.9, M53.0, M53.1, M53.2X7, M53.2X8, M53.3, M53.82, M53.9, M54.02, M54.08, M54.10, M54.12, M54.13, M54.14, M54.15, M54.16, M54.17, M54.2, M54.30, M54.5, M54.6, M54.89, M54.9, M60.009, M60.10, M60.20, M60.9, M61.00, M61.10, M61.40, M61.59, M61.9, M62.00, M62.10, M62.3, M62.40, M62.50, M62.838, M62.84, M62.89, M62.89, M62.9, M65.00, M65.20, M65.30, M65.4, M65.80, M65.80, M65.849, M65.879, M65.9, M66.10, M66.18, M66.239, M66.249, M66.259, M66.269, M66.339, M66.349, M66.369, M66.829, M66.879, M66.88, M66.9, M67.00, M67.40, M67.41, M67.42, M67.43, M67.44, M67.45, M67.46, M67.50, M67.80, M67.88, M67.90, M70.039, M70.1x, M70.2x, M70.3x, M70.40, M70.5x, M70.6x, M70.7x, M70.98, M71.00, M71.20, M71.30, M71.30, M71.40, M71.50, M71.80, M71.9, M72.0, M72.1, M72.2, M72.4, M72.6, M72.9, M75.0x, M75.10x, M75.120, M75.2x, M75.3, M75.3x, M75.4x, M75.5x, M75.8x, M75.8x, M76.10, M76.20, M76.40, M76.50, M76.60, M76.829, M76.899, M77.00, M77.10, M77.20, M77.30, M77.40, M77.50, M77.8, M77.9, M79.0, M79.1, M79.2, M79.3, M79.4, M79.5, M79.609, M79.7, M79.81, M79.89, M79.9, M80.x, M81.x, M84.x, M85.x, M86.1x, M86.2x, M86.6x, M86.9, M86.9, M87.x, M88.9, M89.x, M89.00, M89.30, M89.38, M89.40, M89.6x, M89.70, M89.8X8, M89.8X9, M90.60, M90.80, M90.8x, M91.80, M92.30, M92.40, M92.50, M92.60, M92.70, M92.8, M93.003, M93.1, M93.20, M93.80, M93.90, M94, M94.0, M94.8X9, M95.x, M96.1, M96.2, M96.3, M96.4, M96.5, M99, M99.83, M99.84, R25.2, R29.898, S02.91XK, S02.91XK, S02.92XK, S12.000K, S12.001K, S12.100K, S12.101K, S12.200K, S12.201K, S12.300K, S12.301K, S12.400K, S12.401K, S12.500K, S12.501K, S12.600K, S12.601K, S22.9XXK, S32.9XXK, S42.009K, S42.009P, S42.209K, S42.209P, S42.90XK, S42.90XP, S52.90XK, S52.90XM, S52.90XN, S52.90XP, S52.90XQ, S52.90XR, S62.90XK, S62.90XP, S72.90XK, S72.90XM, S72.90XN, S72.90XP, S72.90XQ, S72.90XR, S82.009P, S82.009Q, S82.009R, S82.90XK, S82.90XM, S82.90XN, S82.90XP, S82.90XQ, S82.90XR, S92.819K, S92.819P, S92.909K, S92.909P, S92.919K, S92.919P, S99.209K, S99.209P, S99.219K, S99.219P, S99.229K, S99.229P, S99.239K, S99.239P, S99.249K, S99.249P, S99.299K, S99.299P |
| Neck pain | M43.6, M47.12, M47.812, M48.02, M50.00, M50.20, M50.30, M50.80, M50.90 , M51.44, M51.45, M53.0, M53.1, M53.82, M54.02, M54.12, M54.13, M54.2, M67.88, M96.1 , S11.90XA, S11.90XA, S11.90XA, S11.90XA, S11.90XA, S11.90XA, S11.90XA , S11.90XA , S11.90XA , S13.101A, S13.101A, S13.111A, S13.121A, S13.131A, S13.141A, S13.151A, S13.161A , S13.171A, S13.181A, S13.4XXA , S13.8XXA |
| Opioid use disorders | F11.1x, F11.2x |
| Other pain conditions | M67.47, M70.30, D48.1, H57.13, H60.509, H60.519, H60.529, H60.539, H60.549, H60.559, H60.599, H60.60, H60.8X1, H60.90, H66.90, H69.80, H92.09, J34.2, K04.4, K04.7, K08.9, K60.3, K60.4, K60.5, K62.89, L60.0, L72.3, M12.30, M12.40, M20.10, M21.61, M21.62, M24.20, M25.70, M25.729, M35.3, M35.7, M54.10, M60.009, M60.10, M60.20, M60.9, M61.00, M61.10, M61.40, M61.59, M61.9, M62.00, M62.10, M62.3, M62.40, M62.50, M62.838, M62.84, M62.89, M62.9, M65.00, M65.20, M65.30, M65.4, M65.80, M65.849, M65.879, M65.9, M66.10, M66.18, M66.239, M66.249, M66.259, M66.269, M66.339, M66.349, M66.369, M66.829, M66.879, M66.88, M66.9, M67.00, M67.40, M67.41, M67.42, M67.43, M67.44, M67.45, M67.46, M67.50, M67.80, M67.88, M67.90, M70.039, M70.1x, M70.2x, M70.3x, M70.40, M70.5x, M70.6x, M70.7x, M70.98, M71.00, M71.20, M71.30, M71.30, M71.40, M71.50, M71.80, M71.9, M72.0, M72.1, M72.2, M72.4, M72.6, M72.9, M75.0x, M75.10x, M75.120, M75.2x, M75.3, M75.3x, M75.4x, M75.5x, M75.8x, M75.8x, M76.10, M76.20, M76.40, M76.50, M76.60, M76.829, M76.899, M77.00, M77.10, M77.20, M77.30, M77.40, M77.50, M77.8, M77.9, M79.0, M79.1, M79.2, M79.3, M79.4, M79.5, M79.609, M79.7, M79.81, M79.89, M79.9, N45.1, N45.2, N45.3, N64.4, N64.51, N64.52, N64.53, N64.59, R07.1, R07.81, R25.2, R29.898, S02.5XXA, S02.5XXB, S03.9XXA, S23.41XA, S29.019A, S39.011A, T14.90XA |
| Pulmonary circulation disorders | I26.x, I27.x, I28.0, I28.8, I28.9 |
| Rheumatoid arthritis/collagen vascular diseases | L94.0, L94.1, L94.3, M05.x, M06.x, M08.x, M12.0, M12.3, M30.x, M31.0 - M31.3, M32.x - M35.x, M45.x, M46.1, M46.8, M46.9 |
